# Supplementary material for: Epstein-Barr Virus (EBV) Is Mostly Latent and Clonal in Angioimmunoblastic T Cell Lymphoma (AITL)
Source: Cancers (Basel). 2022 Jun 12;14(12):2899. doi: 10.3390/cancers14122899 (PMC9221046; doi:10.3390/cancers14122899)
Supplement: Supplementary file 1 [file cancers-14-02899-s001.zip › cancers-1699745-supplementary.pdf]

cancers MDPI

Supplementary Material

Epstein-Barr Virus (EBV) Is Mostly Latent and Clonal in Angioimmunoblastic T Cell Lymphoma (AITL)

Racha Bahri, François Boyer, Mohamad Adnan Halabi, Alain Chaunavel, Jean Feuillard, Arnaud Jaccard and Sylvie Ranger-Rogez *

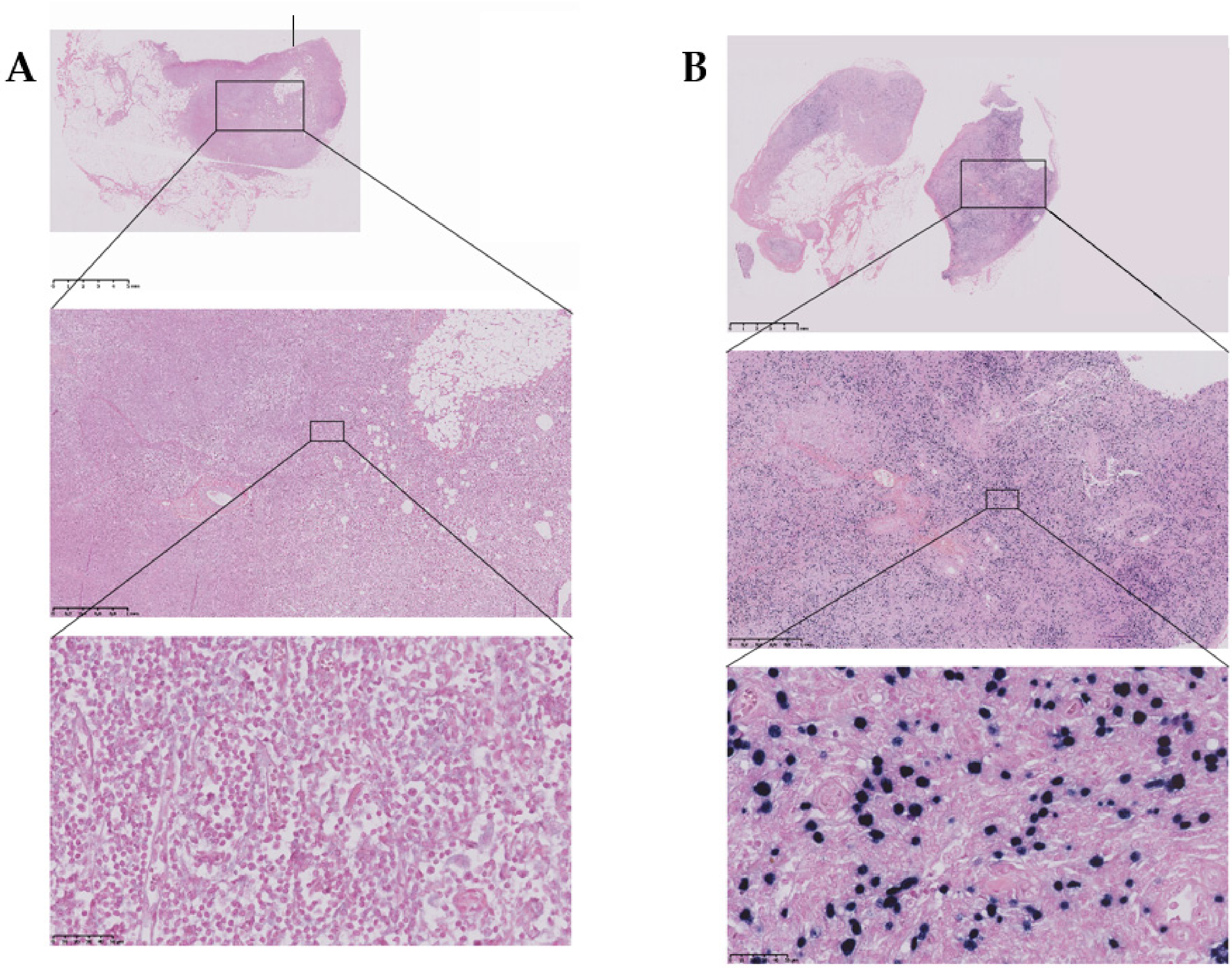

Figure S1. Results obtained for EBER ISH staining. (**A**) Negative staining for control 1 (**B**) Positive staining for AIL8 patient for example.

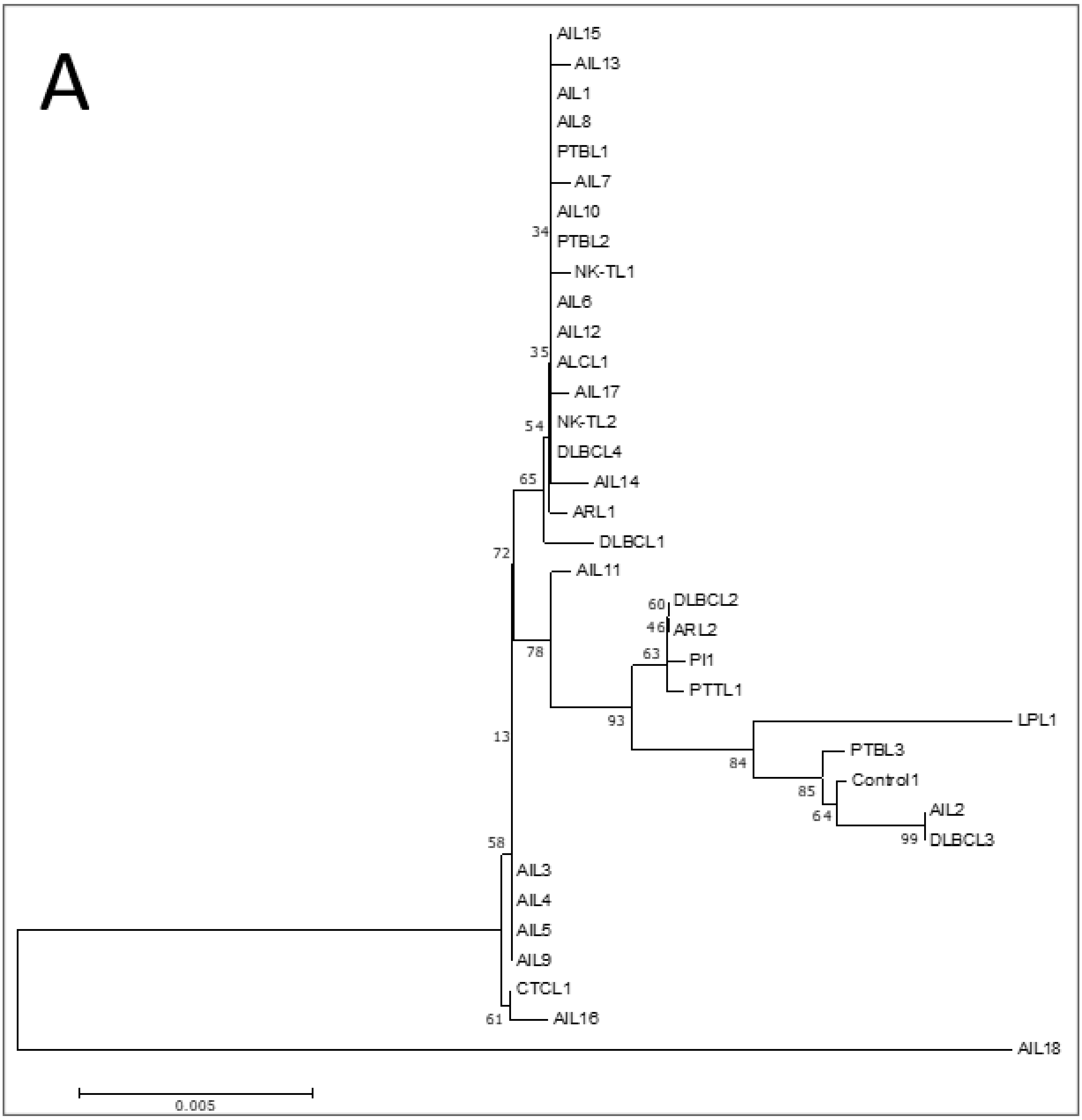
A
AIL15
AIL13
AIL1
AIL8
PTBL1
AIL7
AIL10
34
PTBL2
NK-TL1
AIL6
AIL12
35
ALCL1
AIL17
54
NK-TL2
DLBCL4
65
AIL14
ARL1
DLBCL1
72
AIL11
60
DLBCL2
46
ARL2
63
PI1
78
PTTL1
93
LPL1
13
84
PTBL3
85
Control1
64
AIL2
99
DLBCL3
58
AIL3
AIL4
AIL5
AIL9
CTCL1
61
AIL16
AIL18
0.005

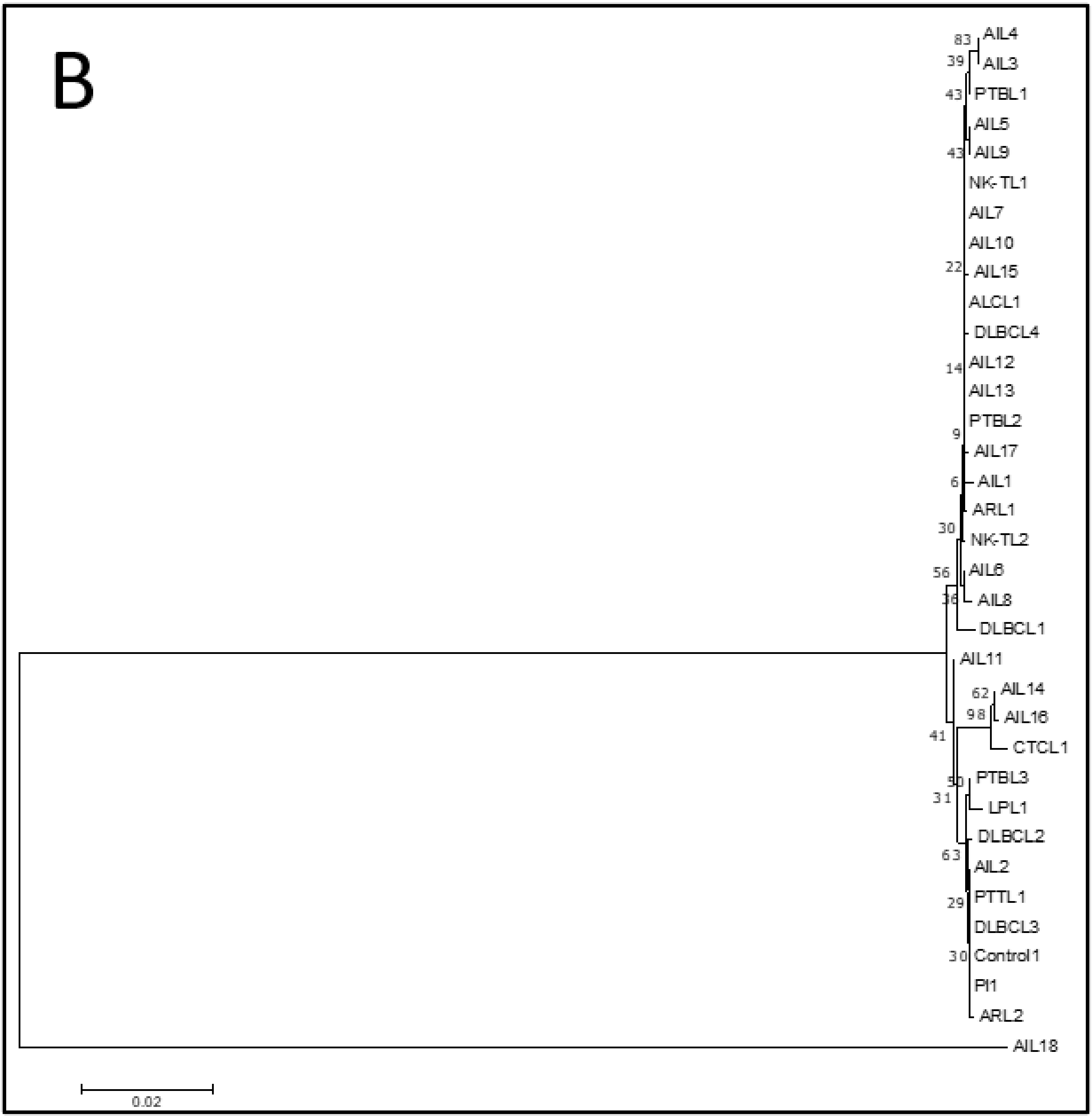
B
AIL4
AIL3
PTBL1
AIL5
AIL9
NK-TL1
AIL7
AIL10
AIL15
ALCL1
DLBCL4
AIL12
AIL13
PTBL2
AIL17
AIL1
ARL1
NK-TL2
AIL6
AIL8
DLBCL1
AIL11
AIL14
AIL16
CTCL1
PTBL3
LPL1
DLBCL2
AIL2
PTTL1
DLBCL3
Control1
PI1
ARL2
AIL18
0.02

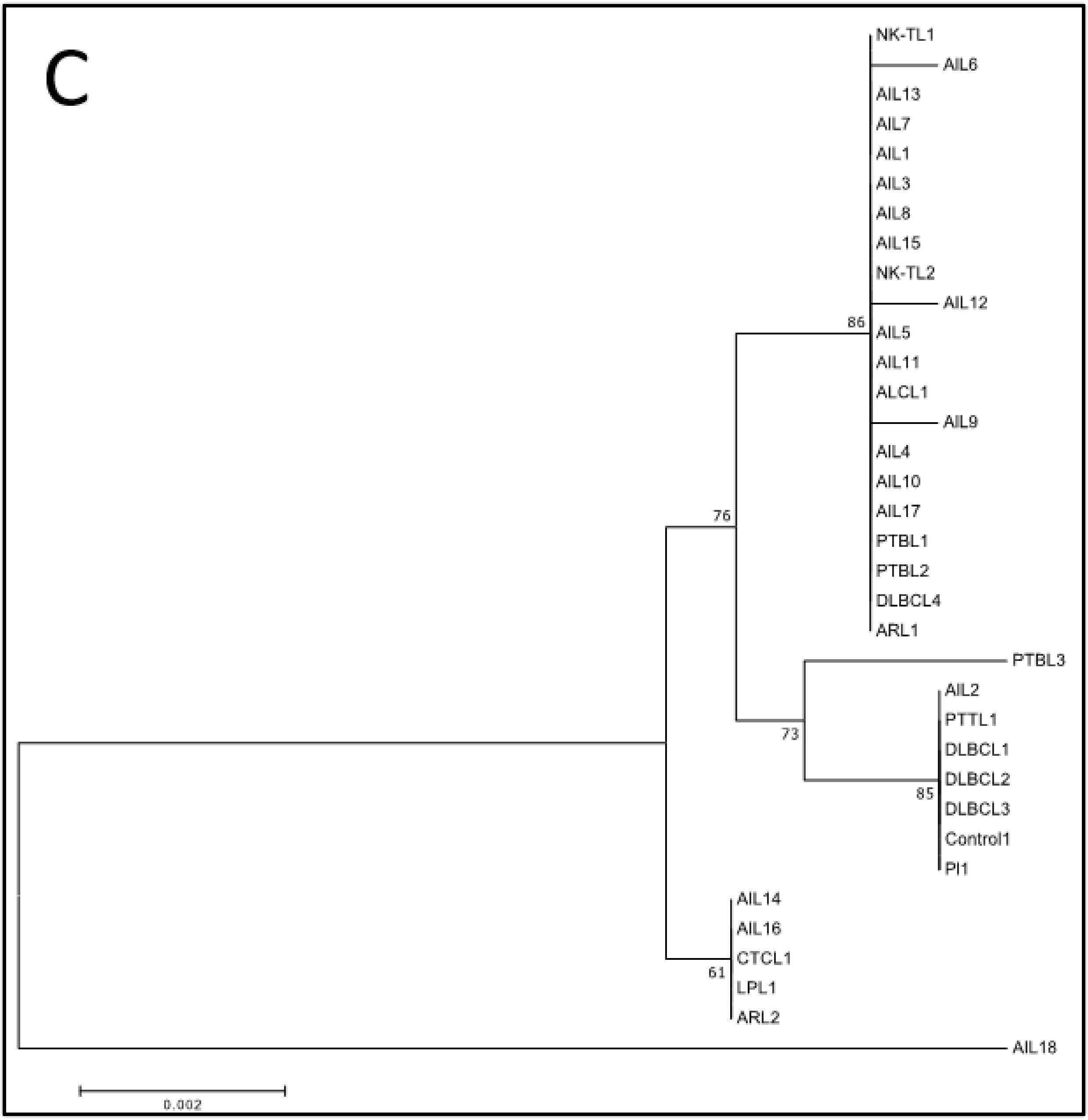

Figure S2. Phylogenetic tree obtained after nucleotide sequence alignment of the different strains. (A) EBNA-1, (B) EBNA-2, (C) EBNA-LP.

Table S1. Genetic Variations and Heterogeneity Determination for All Samples.

Samples	AIL1	AIL2	AIL3	AIL4	AIL5	AIL6	AIL7	AIL8	AIL9	AIL10	AIL11	AIL12	AIL13	AIL14	AIL15	AIL16	AIL17
**All**	756	1058	904	861	919	606	892	612	861	615	725	647	625	636	660	948	664
**SNPs**	727	1010	872	829	877	580	846	589	833	593	699	626	601	606	630	910	639
**Insertions**	15	28	17	18	25	17	30	16	15	14	16	11	15	20	18	23	16
**Deletions**	14	20	15	14	17	9	16	7	13	8	10	10	9	10	12	15	9
**Protein-coding**	539	807	673	641	650	423	664	431	631	428	546	453	450	431	481	733	465
**Protein-coding SNPs**	532	788	664	630	641	414	644	423	625	421	536	447	441	420	467	716	455
**Synonymous SNPs**	290	415	348	324	342	211	334	214	329	211	280	226	229	223	231	396	227
**Non-synonymous SNPs**	242	373	316	306	299	203	310	209	296	210	256	221	212	197	236	320	228
**Protein-coding insertions**	4	11	3	6	5	6	13	5	3	3	5	3	5	6	9	10	7
**Protein-coding deletions**	3	8	6	5	4	3	7	3	3	4	5	3	4	5	5	7	3
**Heterogeneity**	52	293	56	169	59	26	75	28	43	34	268	36	26	35	46	45	49

Samples	AIL18	ALCL1	CTCL1	NK-TL1	NK-TL2	PTTL1	PTBL1	PTBL2	PTBL3	PTBL4	DLBCL1	DLBCL2	DLBCL3	DLBCL4	LPL1	ARL1	ARL2	Control1	PI1
**All**	1059	768	813	1095	644	1282	647	706	2022	1328	530	1236	1097	649	1787	1872	1258	1769	1696
**SNPs**	1030	730	781	1050	609	1238	619	670	1932	1267	505	1169	1043	615	1739	1816	1214	1712	1637
**Insertions**	18	20	22	32	22	25	17	25	54	38	14	41	32	21	25	34	23	34	37
**Deletions**	11	18	10	13	13	19	11	11	36	23	11	26	22	13	23	22	21	23	22
**Protein-coding**	785	544	588	858	456	1008	457	481	1614	1033	385	972	893	471	1393	1540	1011	1358	1321
**Protein-coding SNPs**	783	529	578	833	441	995	444	467	1568	1011	376	943	865	457	1382	1514	995	1339	1300
**Synonymous SNPs**	445	278	312	362	225	538	230	234	829	541	192	488	486	230	766	714	539	746	637
**Non-synonymous SNPs**	338	251	266	471	216	457	214	233	739	470	184	455	379	227	616	800	456	593	663
**Protein-coding insertions**	2	8	7	21	8	7	8	8	29	13	5	19	19	10	5	17	8	14	14
**Protein-coding deletions**	0	7	3	4	7	6	5	6	17	9	4	10	9	4	6	9	8	5	7
**Heterogeneity**	139	25	83	56	36	793	32	59	1175	700	94	210	129	49	382	186	75	824	602
